# *Swertia hongquanii*, a new species of Gentianaceae from Mt. Wuling, southern China

**DOI:** 10.3897/phytokeys.132.37009

**Published:** 2019-09-19

**Authors:** Jiaxiang Li, Yongfu Xu, Lijuan Zhao

**Affiliations:** 1 Faculty of Forestry, Central South University of Forestry and Technology, Changsha, 410004, Hunan, China Central South University of Forestry and Technology Changsha China; 2 Faculty of Life Science and Technology, Central South University of Forestry and Technology, Changsha 410004, Hunan, China Central South University of Forestry and Technology Changsha China

**Keywords:** *
Swertia
*, new taxon, limestone cliffs, Mt. Tianmen

## Abstract

*Swertia hongquanii* Jia X. Li, a new species of Gentianaceae, is described and illustrated from Mt. Wuling, southern China. It grows on the tops of steep limestone mountains surrounded by cliffs above an altitude of ca. 1400 m. This species resembles *Swertia bimaculata*, but differs from the latter by the stem leaf blades 2.0–4.5 × 1.0–2.5 cm, ovate to ovate-cordate, base cordate and subamplexicaul, the seeds irregularly polyhedral and the seed coats minutely thorny and reticulate. Based on morphological traits, i.e. the inflorescence structure and the number and structure of the nectaries, the new species may be a member of series *Maculatae*. A key to the species of series of section MaculataeOphelia is provided.

## Introduction

Mt. Wuling is located at the eastern edge of the Yunnan-Guizhou Plateau in southern China. This plateau has an ancient geological history and complex terrain and geomorphology (Zhu et al. 2015). Due to its long history and changing paleogeography, this area presents a great variety of physiographical characteristics and abundant diverse ecosystems within a short extent of space. Additionally, it has become an important channel for the concentration, diffusion and migration of east Asiatic angiosperms (Chen et al. 2002; Li et al. 2008; Li and Yu 2014; Zhu et al. 2015). Mt. Wuling is rich in flowering plants (4083 species: Chen et al. 2002) and several new taxa have been described from this area recently (e.g. Peng et al. 2007; Li and Yu 2014; Zhang et al. 2015; Liu et al. 2016). Here we propose another new species of *Swertia* L. (Gentianaceae), collected from Tianmenshan National Forest Park in this area.

*Swertia* consists of 3 subgenera, 11 sections and over 160 species (Joshi 2011; Rybczynski et al. 2014; Ho and Liu 2015). The genus is easily recognisable by the rotate corolla and by the presence of coralline nectariferous glands (Suksathan 2001; Ho and Liu 2015). The genus shows a North Temperate and South Temperate disjunctive (Pan-temperate) distribution pattern (Ho and Liu 2015). The majority of species are centred in Asia. South-western China and adjacent regions are diversity centres and initial diversification centres for this genus (Ho et al. 1994; Ho and Liu 2015). In China, approximately 75 species occur, mainly in mountains at an elevation above 1000 m (Ho and Pringle 1995). Moreover, several new species have been described from China since the account of the genus for the Flora of China was published (e.g. Chen et al. 2008; Ho and Liu 2010, 2015; Chen et al. 2016).

The new species from Mt. Wuling first came to our attention in September 2007 during our plant investigation in Tianmenshan National Forest Park. It was initially misidentified as *Swertia bimaculata* (Siebold & Zucc.) Hook.f. & Thomson ex C.B.Clarke due to its lax panicles of cymes and two naked nectaries per corolla lobe. In 2016, in the course of digitising the specimens in CSFI, we found a unique specimen, collected from Mt. Tianmen by Mr. Hongquan Huang (13050406) on 20 September 2013. It was identified as *Swertia bimaculata* but differs by its petite shape and cordate leaf base. Subsequently, we again visited Tianmenshan National Forest Park to observe this plant in September and November 2017 and collected more flowering and fruiting material. After a morphological comparison, the specimens from Tianmenshan National Forest Park are confirmed to be an undescribed species of *Swertia*, probably belonging to series Maculatae T.N.Ho & S.W.Liu in section Ophelia (Griseb.) Gilg in subgenus Ophelia (Griseb.) C.B.Clarke.

## Materials and methods

During three field expeditions in September 2007, September and November 2017, a total of fifteen flowering and five fruiting individuals from the type locality were collected from Tianmenshan National Forest Park, Zhangjiajie City, Hunan Province, Southwest China. The information and measurements of the new species were taken from live and dried herbarium specimens and from field data. Seeds were examined and imaged with a Leica M205C stereomicroscope attached to a video camera (Leica DFC495). The morphological comparisons with related species, viz., *Swertia bimaculata*, *S. tashiroi* Makino, *S. oculata* Hemsl., *S. tozanensis* Hayata, *S. cordata* (Wall. ex G.Don) C.B.Clarke and *S. shintenensis* Hayata, are based on herbarium specimens (about 2300 specimens) and relevant literature (Ho et al. 1988; Ho and Pringle 1995; Rybczynski et al. 2014; Ho and Liu 2015). Specimens deposited in the following herbaria were examined: CSFI, CSH, CZH, JIU, HTC, IBK, IBSC, LBG, KUN, PE, SYS and WUK (Thiers 2015).

The number of mature individuals was recorded in the field in twenty 1 m^2^ sampling plots. We assessed the preliminary conservation status of the new species using our field knowledge and applying the IUCN (2017) criteria. The taxonomic treatment of the genus *Swertia* follows Ho and Liu (2015).

## Taxonomic treatment

### Swertia hongquanii

Taxon classificationPlantaeGentianalesGentianaceae

Jia X. Li sp. nov.

89EA9950-D325-5665-AAF9-2F365958E922

urn:lsid:ipni.org:names:60479372-2

Figs 1 , 2 , 3

#### Diagnosis.

The new species is similar to *Swertia bimaculata*, but differs from the latter by its leaf blades ovate to ovate-cordate, 2.0–4.5 × 1.0–2.5 cm with base cordate and subamplexicaul (vs. broadly elliptic to ovate-lanceolate, 3.5–9 × 1.0–4 cm with base tapered to obtuse), and its seeds irregularly polyhedral with minutely thorny and reticulate seed coat (vs. globose with seed coat finely warty) (Table 1).

#### Type.

CHINA. Hunan province: Zhangjiajie City, Mt. Tianmen, 29°3'N, 110°28'E, elev. 1400 m, 23 September 2016, *J. X. Li 092502* (holotype CSFI, bar code: CSFI063656; isotypes CSFI, IBSC, PE).

#### Description.

Biennial herbs, 2–40 cm tall. Roots yellow, fibrous. Stems erect, sometimes branched from the lower part, subquadrangular, 1–3 mm in diam., with narrow wings on angles. Basal leaves quickly withering, blades elliptic to obovate, 1–3.5 × 0.8–3 cm, apex obtuse, base cuneate and decurrent, veins yellow-white, distinct, pinnate; petioles flattened, winged, ca. 0.5–2.5 cm long. Stem leaves sessile or shortly petiolate, leaf blades ovate to ovate-cordate, apex acute, base cordate and subamplexicaul, 2.0–4.5 × 1.0–2.5 cm, smaller towards stem apex, veins 3–5, arcuate, distinct, green or yellow-green.

Inflorescence a panicle of cymes, lax, 5–20(–25) × 4–20 cm, few- or many-flowered; axes spreading. Flowers (4-)5-merous. Pedicels spreading to erect, slender, subquadrangular, 0.6–4 cm. Calyx 1/2 to 2/3 as long as corolla, tube 1–2 mm long, lobes narrowly elliptic, 3–6 mm long, with 3 slender and distinct veins, apex acute. Flowers to 2.0 cm in diam.; corolla white with purple spots on the upper half of the lobes adaxially but less visible abaxially, tube 1–2 mm long; lobes elliptic-lanceolate, 0.5–1.0 × 0.2–0.4 cm, widest at the middle, apex acuminate to acute. Nectaries 2 per corolla lobe, situated in the middle of corolla lobe, semi-circular, reduced to a naked gland patch without raised margin, yellow-green. Stamens with filaments ca. 4 mm long, white; anthers ellipsoid, ca. 1.5 mm long, purple. Style short, ca. 0.5 mm long; stigma lobes capitate. Capsules narrowly ovoid, to 1.3 cm long. Seeds polyhedral, compressed irregularly, with distinct angles, ca. 1.0 × 0.5 mm, dark brown; seed coat minutely thorny and reticulate.

**Figure 1.**
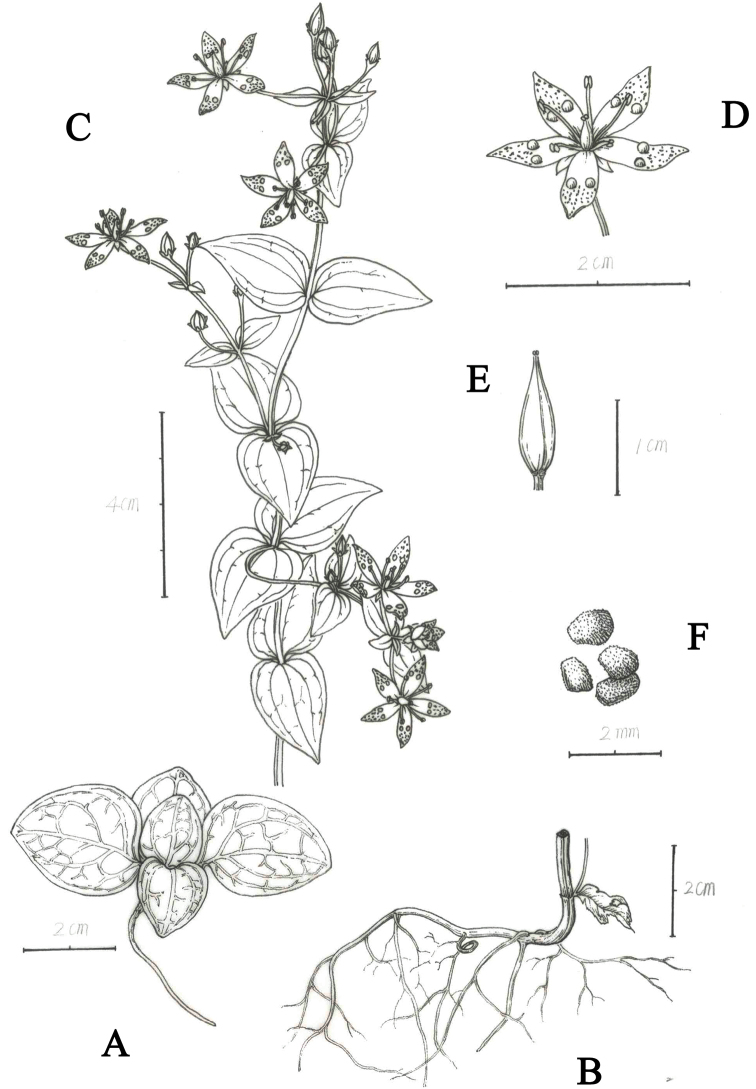
*Swertia hongquanii***A** seedlings showing basal leaves **B** root **C** flowering plant **D** flower showing corolla, nectaries, stamens and pistil **E** capsule **F** seeds. Drawn by Jing Tian.

#### Phenology.

Flowering was observed in September and October. Fruiting was only observed in November, but probably extends till December.

#### Distribution and habitat.

*Swertia hongquanii* is known only from the type location in Tianmenshan National Forest Park. The climate here is cool, foggy and humid (annual rainfall ca. 1700 mm) and belongs to the subtropical monsoon climate (Li et al. 2008; Zhang et al. 2015). The new species grows on the tops of steep limestone mountains surrounded by cliffs above an altitude of ca. 1400 m. The clifftops are covered by mixed evergreen-deciduous forest dominated by species of Fagaceae, Lauraceae, Betulaceae, Aceraceae and Ulmaceae (Li et al. 2008). One population occurs in the crevices of limestone cliffs; it is covered by shrubs and herbs with little soil but is rich in humus. It is accompanied by *Viola davidii* Franch., *Youngia* Cass. sp., *Thalictrum ichangense* Lecoy. ex Oliv., *Aster ageratoides* Turcz., *Calamagrostis arundinacea* (L.) Roth, *Carex filicina* Nees, *Carex* L. sp., *Leptopus chinensis* (Bunge) Pojark., *Zanthoxylum bungeanum* Maxim., Rubus innominatus S.Moore var. kuntzeanus (Hemsl.) L.H.Bailey, *Rubus henryi* Hemsl. & Kuntze, *Salix mictotricha* C.K.Schneid., *Carpinus dayongiana* K.W.Liu & Q.Z.Lin and *Betula chinensis* Maxim. Another population grows in the herb layer of forest dominated by *Quercus multinervis* (W.C.Cheng & T.Hong) Govaerts and *Hovenia dulcis* Thunb. with deep and fertile soil. Other herbaceous species are *Ophiopogon japonicus* (Thunb.) Ker Gawl., *Carex gibba* Wahlenb., *Sanicula orthacantha* S.Moore, *Pimpinella diversifolia* DC. and *Viola diffusa* Ging.

#### Etymology.

The species is named after Mr. Huang Hongquan for his help during our field investigation. He was also the first to collect this new species.

**Figure 2.**
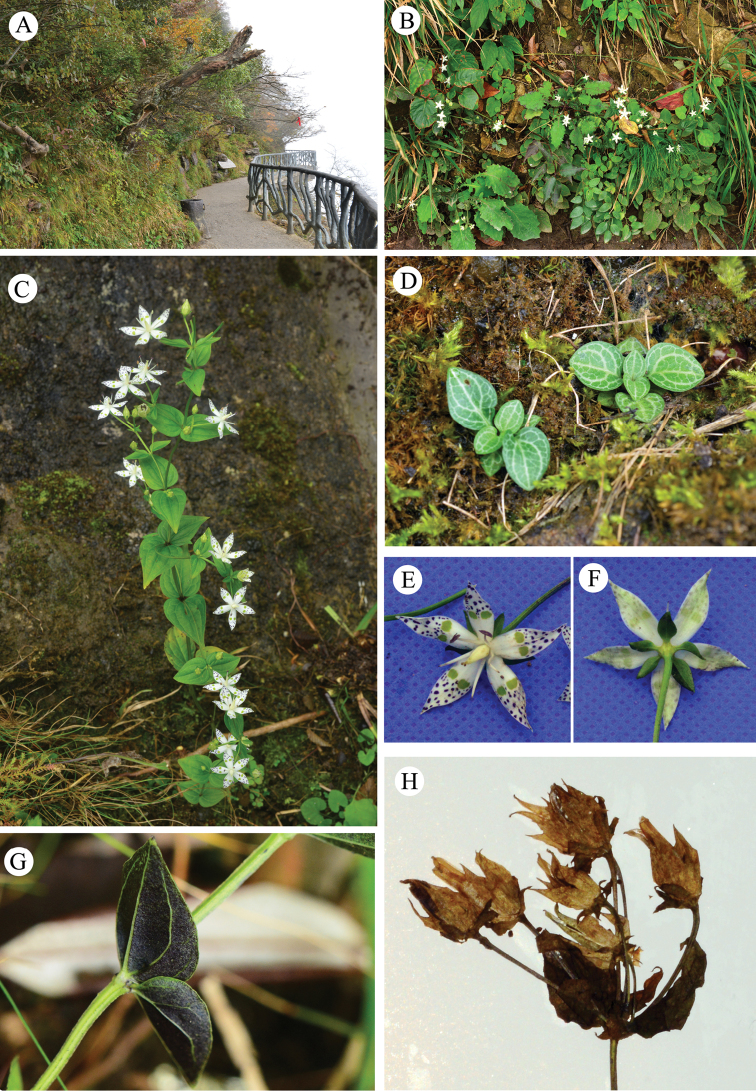
Habitat and morphology of *Swertia hongquanii*. **A–B** Habitat **C** flowering plant **D** seedlings showing basal leaves **E** adaxial view of flower showing calyx, corolla, nectaries, stamens and pistil **F** abaxial view of flower showing calyx and corolla **G** stem with a pair of leaves **H** flowering and fruiting plant; centrally, a capsule with persistent corolla can be seen. Photos: Jiaxiang Li.

#### Local name.

Tianmenshan Zhang Ya Cai

#### Conservation status.

Despite several investigations in Mt. Tianmen and the surrounding areas (96 km^2^), two populations with nearly 500 individuals each (total < 1000) of *Swertia hongquanii* were found only at the type locality (towering summit terrace with an area of 2 km^2^). Presently, a tourist plank walkway passes through this location and its habitat could be easily disturbed or destroyed. According to the IUCN (2017) criterion D thresholds (mature individuals < 1000, AOO < 20 km^2^), the new species could be assessed as VU. However, it grows in the upper part of steep limestone mountains surrounded by cliffs, which previously made it impossible to be encountered until a plank walkway was built across the cliff face for tourists. With limited fieldwork executed at present, it is possible that more populations could be found in similar habitats of the Wuling mountain areas. Therefore, we consider the species DD (Data Deficient).

#### Additional specimens examined.

CHINA: Hunan, Zhangjiajie City, Mt. Tianmen, 20 September 2013, 29°3'N, 110°28'E, limestone, 1400 m alt., *Hongquan Huang* 13050406 (CSFI); the same locality, 20 November 2017, *Hongquan Huang* HHQ02 (CSFI); the same locality, 25 September 2017, J.X. Li 092503 (CSFI).

**Figure 3.**
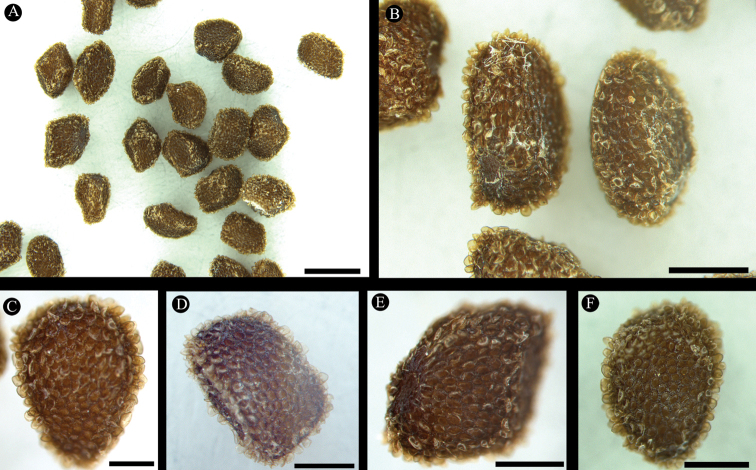
Seeds of *Swertia hongquanii*. Scale bars: 1 mm (**A**); 0.5 mm (**B**); 0.2 mm (**C**); 0.3 mm (**D–F**). Photos: Jiaxiang Li.

## Discussion

Morphologically, *Swertia hongquanii* resembles *S. bimaculata* in possessing two naked gland patches in the middle of each corolla lobe, whereas it is clearly distinguished by stem leaf and seed characteristics (Table 1). During field investigations, we observed that most *S. bimaculata* flowers were withered while the new species was just beginning to blossom in populations in the same locality (separated by a distance of ca. 300 m). We therefore infer that the morphological differences with *S. bimaculata* are interspecific rather than intraspecific variations driven by ecological conditions.

From its overall vegetative appearance, *Swertia hongquanii* also resembles *S. cordata*, with both species having slender and subquadrangular stems with wings on the angles, ovate to ovate-cordate stem leaves with cordate to subamplexicaul bases. *Swertia hongquanii* is clearly distinguished from *S. cordata*, however, by the two nectaries in the middle of the corolla lobes; *S. cordata*, in contrast, has a single nectary on the base of the corolla lobe (Table 1) (Ho and Liu 2015).

**Table 1.** Morphological comparison of *Swertia hongquanii*, *S. bimaculata*, and *S. cordata* (adapted from Ho and Liu 2015).

	*S. hongquanii*	*S. bimaculata*	*S. cordata*
Habit	biennial	annual or biennial	annual
Height	2–40 cm tall	30–140(–200) cm tall	15–40(–80) cm tall
Stem	1–3 mm in diam., simple or branched from the lower part	2–6 mm in diam., branched from the middle	1.5–2 mm in diam., branched at base or from the middle
Stem leaves	ovate to ovate-cordate, base cordate and subamplexicaul, 2.0–4.5 × 1.0–2.5 cm	broadly elliptic to ovate-lanceolate, base narrowly tapered to obtuse, 3.5–9 × 1–4(–5) cm	ovate to ovate-cordate, base cordate and subamplexicaul, 0.8–2.3 × 0.5–1.2 cm
Inflorescence	lax, axes and pedicels spreading, few or many-flowered, 5–20(–25) × 4–20 cm	lax, axes and pedicels spreading, many-flowered, 10–50 × 10–30 cm	compact, axes and pedicels not spreading, dense, many-flowered, 3–30(–40) × 2–10 cm
Pedicel length	0.6–4 cm	0.6–4 cm	0.3–1 cm
Corolla	white with purple spots, ca.20 mm in diam.	yellow or white, with purple spots, to 25 mm in diam.	pale purple, with dark purple veins, 10–15 mm in diam.
Nectaries	2 per corolla lobe, yellow-green, semi-orbicular, in the middle of corolla lobes	2 per corolla lobe, yellow-green, orbicular, in the middle of corolla lobes	1 per corolla lobe, yellow, rhomboid to orbicular, at the base of corolla lobes
Seeds	polyhedral, ca. 1.0 mm × 0.5 mm, seed coat minutely thorny and reticulate	globose, 1–1.5 mm in diam., seed coat finely warty	ellipsoid to globose, 0.8-1 mm in diam., seed coat longitudinally and thinly corrugate

According to the classification of Ho and Pringle (1995) and Ho and Liu (1980; 2015), the species of series Maculatae of section Ophelia of subgenus Ophelia are distinguished by their stems being strongly branched, their inflorescences being panicles of cymes and by the presence of one or two nectaries on each corolla lobe reduced to a naked gland patch without raised margin. Six species, viz., *S. bimaculata*, *S. oculata*, *S. cordata*, *S. tozanensis*, *S. shintenensis* and *S. tashiroi* were recognised as members of series. *Maculatae*, distributed in Asia (Ho and Liu 2015). Based on morphological traits, especially those of inflorescences and nectaries, the new species may also be a member of series *Maculatae* (Figures 1–3).

The seeds of *S. hongquanii* are easily distinguished from other species of series *Maculatae*, as they are irregular polyhedrons with minutely thorny and reticulate seed coats (Figure 3), whereas those of the other species of series *Maculatae* are ellipsoid to globose with finely warty seed coats (*S. tashiroi*, *S. shintensis*, *S. bimaculata*, *S. tozanensis* and *S. oculata*) or with longitudinally and thinly corrugate seed coats (*S. cordata*) (Ho and Liu 2015).

### Key to species of series Maculatae (adapted from Ho and Liu 2015)

**Table:** 

1	Nectaries one per corolla lobe	**2**
–	Nectaries two per corolla lobe	**3**
2	Basal leaves quickly withering; inflorescences usually narrow and dense; corolla pale purple; nectaries on base of corolla lobe; seed coat longitudinally and thinly corrugate	***S. cordata***
–	Basal leaves persistent; inflorescences rounded and lax; corolla yellow or yellow-green; nectaries in the middle of corolla lobe; seed coat finely warty	**4**
3	Leaf blades broadly elliptic to ovate-lanceolate or ovate to ovate-cordate, more than 1 cm wide; corolla with purple spots	**5**
–	Leaf blades linear, linear-lanceolate or lanceolate, 0.2–0.7 cm wide; corolla with yellow-green or dark spots	**6**
4	Upper stem leaves lanceolate to linear; corolla unspotted; seeds 0.5 mm in diam	***S. tashiroi***
–	Upper stem leaves ovate; corolla with purple-brown spots on upper portion; seeds 0.7–1 mm in diam.	***S. shintenensis***
5	Plants 30–140 (–200) cm tall; leaf blades broadly elliptic to ovate-lanceolate, base narrowly tapered to obtuse; seeds globose; seed coat warty	***S. bimaculata***
–	Plants 2–30 cm tall; leaf blades ovate to ovate-cordate, base cordate and subamplexicaul; seeds polyhedral; seed coat minutely thorny and reticulate	***S. hongquanii***
6	Calyx lobes linear to linear-oblong; corolla lobes elliptic-lanceolate, white, with yellow-green spots, apex acuminate and apiculate	***S. oculata***
–	Calyx lobes ovate-lanceolate to spathulate; corolla lobes oblong, pale-yellow, with dark spots, apex obtuse to acute	***S. tozanensis***

## Supplementary Material

XML Treatment for Swertia hongquanii
